# Characterization of Circulating Fibrocytes in People Living with HIV on Stable Antiretroviral Therapy

**DOI:** 10.4049/immunohorizons.2200085

**Published:** 2022-11-01

**Authors:** Logan S. Dean, Dominic C. Chow, Lishomwa C. Ndhlovu, William A. Boisvert, Sandra P. Chang, Cecilia M. Shikuma, Juwon Park

**Affiliations:** * Department of Tropical Medicine, Medical Microbiology, and Pharmacology, University of Hawaii at Manoa, Honolulu, HI;; † Hawaii Center for AIDS, John A. Burns School Medicine, University of Hawaii at Manoa, Honolulu, HI;; ‡ Center for Cardiovascular Research, University of Hawaii at Manoa, Honolulu, HI

## Abstract

Highly effective combination antiretroviral therapy has reduced HIV infection to a manageable chronic disease, shifting the clinical landscape toward management of noninfectious comorbidities in people living with HIV (PLWH). These comorbidities are diverse, generally associated with accelerated aging, and present within multiple organ systems. Mechanistically, immune dysregulation and chronic inflammation, both of which persist in PLWH with well-controlled virally suppressive HIV infection, are suggested to create and exacerbate noninfectious comorbidity development. Persistent inflammation often leads to fibrosis, which is the common end point pathologic feature associated with most comorbidities. Fibrocytes are bone marrow–derived fibroblast-like cells, which emerged as key effector cells in tissue repair and pathologic fibrotic diseases. Despite their relevance to fibrosis, the circulating fibrocyte concentration in PLWH remains poorly characterized, and an understanding of their functional role in chronic HIV is limited. In this study, utilizing PBMCs from a cross-sectional adult HIV cohort study with matched uninfected controls (HIV–), we aimed to identify and compare circulating fibrocytes in blood. Both the percentage and number of fibrocytes and *α*-smooth muscle actin^+^ fibrocytes in circulation did not differ between the HIV+ and HIV‒ groups. However, circulating fibrocyte levels were significantly associated with increasing age in both the HIV+ and HIV‒ groups (the percentage and number; *r* 5 0.575, *p* ≤ 0.0001 and *r* 5 0.558, *p* ≤ 0.0001, respectively). Our study demonstrates that circulating fibrocyte levels and their fibroblast-like phenotype defined as collagen I and *α*-smooth muscle actin^+^ expression are comparable between, and strongly associated with, age irrespective of HIV status.

## INTRODUCTION

The success of antiretroviral therapy (ART) has shifted the clinical management of people living with HIV (PLWH) from HIV/AIDS-defining illnesses to chronic noninfectious comorbidities (NICMs) management (1). Cardiometabolic (2–4), cancer (5), renal (6), hepatic (7–11), neurocognitive (12–14), and pulmonary (15, 16) diseases occur at increased rates among PLWH, independent of viral suppression and ART adherence. A disproportionate incidence of NICMs in PLWH is associated with chronic inflammation, low-grade viral replication, and the increasing age of PLWH (17, 18). These risk factors predispose PLWH to fibrosis, a dysregulated wound-healing response characterized by the excessive deposition of extracellular matrix, eventually leading to organ dysfunction (19). Prevalence of fibrosis in PLWH on stable ART is increased in the heart (20–22) and liver (7, 10, 11) when compared with HIV-uninfected individuals. Lung fibrosis has been noted to increase in PLWH when compared with HIV-uninfected individuals (4, 23); however, an exact correlation with HIV infection, rather than the presence of COPD, is not established.

Fibrocytes are bone marrow–derived fibroblast-like cells found in circulation and within tissues. This unique cell type adopts a spindle shape when adherent and is characterized by simultaneous expression of inflammatory features of macrophages and the tissue remodeling properties of fibroblasts (24). Consistent with their bone marrow origin, fibrocytes express several hematopoietic cell markers, including CD45, CD11b, and CD14 in addition to collagens and glycosaminoglycans, features that are characteristic of fibroblasts (25). Fibrocytes also express chemokine receptors, such as CCR2, CCR3, CCR5, CCR7, and CXCR4, and migrate into areas of inflammation via chemo-taxis (26). These cells possess Ag-presenting functionality and are potent stimulators of CD4^+^ and CD8^+^ T cells (27, 28). Fibrocyte levels within circulation have been shown to increase by 5–10% in patients with fibrotic diseases compared with healthy individuals, and increased numbers of circulating fibrocytes are reported in patients with organ fibrosis across multiple organ systems (8, 29–38). Although it is relatively well known that elevated fibrocyte numbers and activation state are linked to pathologic fibrotic diseases in the general population, to our knowledge, there is limited information regarding their abundance, characteristics, and functionality in PLWH.

In this study, we identified circulating fibrocytes in PBMC fractions from PLWH on stable ART and compared their proportions and activation state with those of HIV‒ individuals who were HIV-seronegative. Our findings provide a foundation for future work to determine whether circulating fibrocyte levels can be potentially used as a biomarker for fibrosis in PLWH and the effects of age.

## MATERIALS AND METHODS

### Study subjects and specimen selection

This cross-sectional study used data and banked PBMCs from the entry time point of longitudinal cohort study H043 (“Role of monocytes/macrophages in HIV-related cardiovascular risk”). The parent protocol was approved by the University of Hawaii Institutional Review Board (protocol ID 2021–00676). Briefly, the study enrolled adults ≥40 y of age with documented HIV infection who were on suppressive ART for >6 mo with plasma HIV RNA <50 copies/ml within 6 mo of screening. Individuals were excluded based on reporting of acute or uncontrolled chronic illness, inability to abide by study requirements, congenital or genetic cardiovascular abnormalities, pregnancy and/or breastfeeding, or those deemed by the principal investigator as unadvisable for study participation. HIV ‒ individuals were confirmed by a negative HIV test within 6 mo of screening and were recruited as a comparator group. Written informed consent was obtained for all participants.

### Clinical parameters

Clinical parameters including height, weight, and blood pressure (BP; systolic and diastolic BP) were measured. T cell subsets and plasma HIV RNA assessment were performed, and undetectable plasma HIV RNA was defined as ≤50 copies/ml. Chemistry and metabolic laboratory results (glucose, insulin, total cholesterol, high-density lipoprotein cholesterol [HDL-C], low-density lipoprotein cholesterol [LDL-C], and triglycerides) were obtained at entry in a fasted state. Participants were assessed for health behaviors (smoking and alcohol intake), medication/drug use, and pre-existing health conditions. Measurement of carotid intima–media thickness was conducted using a previously published protocol (29).

### PBMC isolation

PBMCs were isolated by Ficoll-Paque Plus (GE Healthcare Biosciences, Piscataway, NJ) following the manufacturer’s protocol. Briefly, venous blood samples were diluted with an equal volume of PBS and then 30 ml was layered over 15 ml of Ficoll-Paque Plus. PBMCs were separated by centrifugation at 400 × *g* for 30 min at room temperature (RT). PBMCs were aspirated from the buffy coat, RBCs lysed, and then washed twice in PBS supplemented with 1% FBS. PBMCs were stored at 5 × 10^6^ cells/ml in 1-ml aliquots of freezing solution comprised of 90% FBS and 10% DMSO by volume in a ‒150°C freezer.

### Fluorescence-conjugated Abs

BV711-conjugated anti-human CD45 was purchased from BD Biosciences (East Rutherford, NJ). PE-conjugated anti-mouse α-smooth muscle actin (α-SMA) was purchased from R&D Systems (Minneapolis, MN). BV605-conjugated anti-human CD14, BV421-conjugated anti-human CD16, PE/Cy-7–conjugated anti-human CD11b, and allophycocyanin-conjugated anti-human CD34 were purchased from BioLegend (San Diego, CA). A LIVE/DEAD fixable yellow dead cell stain kit was purchased from Invitrogen (Waltham, MA). FITC-conjugated anti-human collagen type 1 (COL-1) was purchased from MilliporeSigma (St. Louis, MO).

### Flow cytometric analysis

Fibrocytes simultaneously express CD45 and COL-1 and are generally believed to originate from CD14^+^ precursors. Myeloid markers CD11b and CD16 were included to distinguish between the three monocyte subsets. α-SMA was included to identify activated fibrocytes. Cryopreserved PBMCs were thawed at 37°C, with 97–98% viability observed following the thaw. Cells were then washed twice in Dulbecco’s PBS (DPBS) before incubation with LIVE/DEAD fixable yellow dead cell stain (Invitrogen, 1:1000) for 30 min at 4°C, protected from light. Cells were then washed twice in ice-cold DPBS, resuspended in 50 μl of human TruStain FcX (BioLegend, San Diego, CA, 1:200), covered, and incubated at RT for 15 min. Cells were washed twice in 1 ml of ice-cold DPBS supplemented with 1% BSA (flow buffer) and incubated with Abs (CD45, CD11b, CD14, CD16, and CD34) diluted in flow buffer. For intracellular staining, cells were re-suspended in 250 μl of BD Cytofix/Cytoperm (BD Biosciences, East Rutherford, NJ) for 30 min at 4°C, protected from light. Cells were then washed twice with 1 ml of BD Perm/Wash buffer (BD Biosciences, East Rutherford, NJ) before incubation with Abs (COL-1 and α-SMA) at RT for 30 min, protected from light. Cells were washed twice with BD Perm/Wash buffer and resuspended in 200 μl of flow buffer.

The gating strategy for α-SMA^+^ fibrocytes was optimized using cultured fibrocytes (Supplemental Fig. 1). Briefly, PBMCs were cultured in flat-bottom tissue culture plates at 2.5 × 10^6^ cells/ml in fibroblast basal medium 2 (PromoCell, Heidelberg, Germany) supplemented with 1% penicillin/streptomycin (Millipore, Burlington, MA) in a CO_2_ incubator at 37°C. For fibrocyte activation, cultured fibrocytes were serum starved in serum-depleted DMEM (Caisson Labs, Smithfield, UT) for 16 h and then treated with or without recombinant human TGF-β1 (R&D Systems, Minneapolis, MN) at 5 or 20 ng/ml overnight in a CO_2_ incubator at 37°C.

Samples were acquired with identical voltage settings on a LSRFortessa (BD Biosciences, East Rutherford, NJ) with ~1.0 × 10^9^ events collected per sample. Ten microliters of AccuCheckȈcounting beads (Life Technologies, Carlsbad, CA) was added prior to acquisition. Compensation beads (Invitrogen, Waltham, MA) were prepared for accurate compensation controls. Data were analyzed using FlowJo (Tree Star, Ashland, OR) software, and absolute cell counts were determined according to the manufacturer’s protocols.

Circulating fibrocytes are defined as CD45^+^/CD11b^+^/CD14^lo/+^/CD16^+/‒^/COL-1^+^ and differentiated circulating fibrocytes as CD45^+^/CD11b^+^/CD14^lo/+^/CD16^+/‒^/COL-1^+^/α-SMA^+^ cells.

### Statistical analysis

Flow cytometry results were analyzed using a Mann–Whitney-*U* test with a 95% confidence interval for between-group comparisons. Spearman correlations were performed for between-variable associations of fibrocyte populations. A *p* value of <0.05 was considered statistically significant for all tests. Statistical analysis was performed by Prism 9 (GraphPad Software, San Diego, CA).

## RESULTS

### PLWH on stable ART display alterations in monocyte subset frequencies

Participant demographics are detailed in Table I. HIV+ and HIV‒ participants were comparable in terms of sex, ethnicity, and age. Approximately 88% were male, a little more than 60% were White, and the median age was ~59 y. HIV+ participants had a median of 19.5 y of ART with median CD4^+^ T cell counts of 747.5 (601–898) cells/μl. Of the NICMs assessed in this study, the prevalence was comparable between the two groups. There were no significant differences in body mass index, glucose, insulin, total cholesterol, HDL-C, LDL-C, triglycerides, or BP between the groups. Absolute lymphocyte count was significantly increased in the HIV+ group compared with the HIV ‒group, whereas absolute neutrophil count, monocyte count, and platelet count were comparable between HIV+ and HIV ‒groups. We first determined the proportion and number of monocyte subset populations as the source of fibrocytes in the context of HIV infection stable on ART. The percentages of CD11b^+^ cells and total monocytes were lower in the HIV+ group compared with the HIV‒ group (Supplemental Fig. 2A, 2B). However, the numbers of CD45^+^,CD11b^+^, and total monocyte cell counts were not different between the two groups (Supplemental Fig. 3A–C). We next determined the monocyte subsets responsible for the decreased proportions of cells observed in the HIV+ group. We found that the percentages of classical monocytes (CD14^+^/CD16^‒^) and intermediate monocytes (CD14^hi^/CD16^+^) from total CD45^+^ cells were lower in the HIV+ group compared with the HIV‒ group, but that nonclassical (CD14^lo^/CD16^+^) monocyte proportions were comparable (Supplemental Fig. 2C, 2D). However, the numbers of monocyte subsets did not differ between the two groups (Supplemental Fig. 3D–F). Taken together, these findings indicate that despite long-term ART, HIV+ individuals exhibit alterations in monocyte subset frequencies.

### Identification and characterization of circulating fibrocytes in HIV + individuals

**FIGURE 1.**
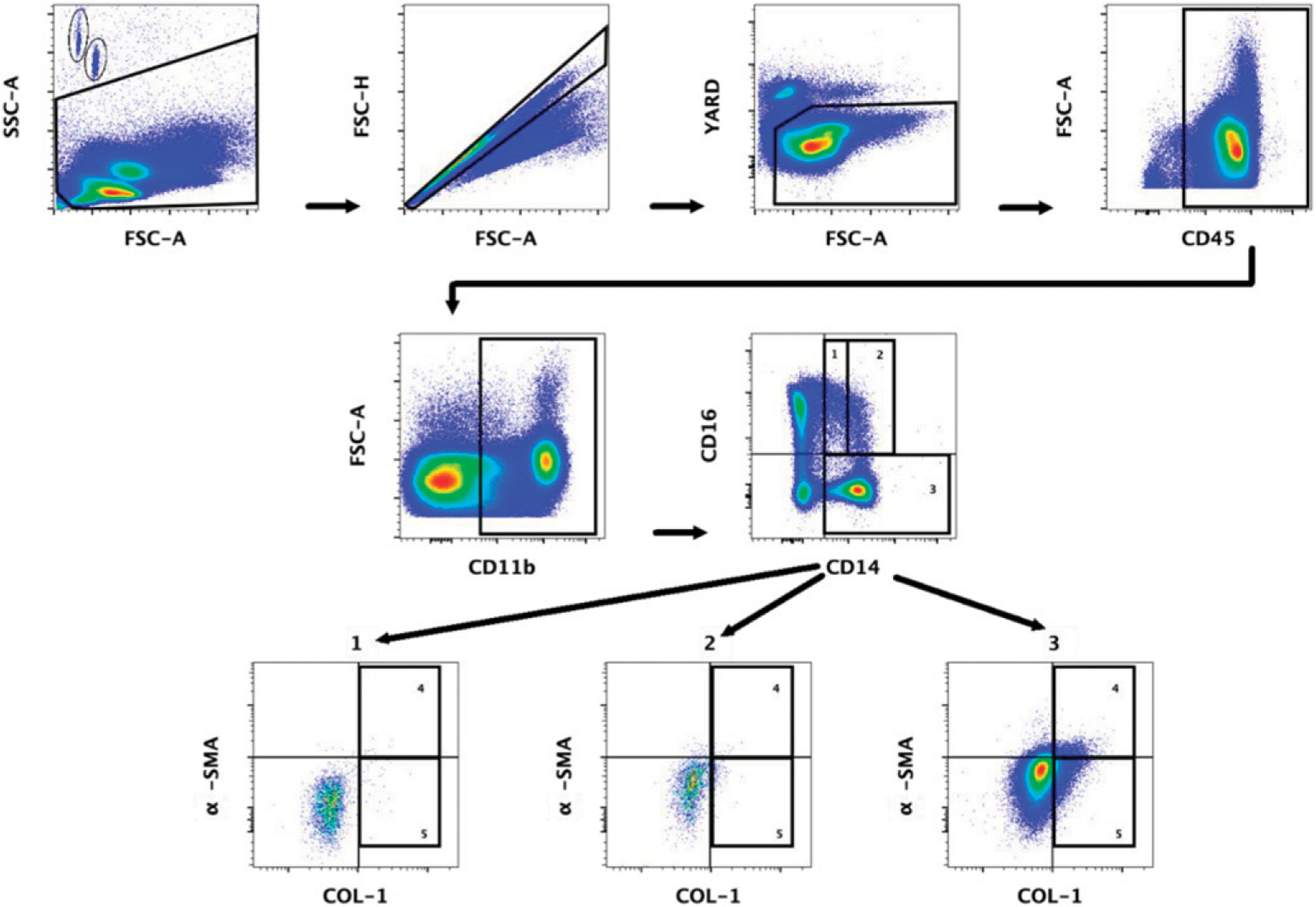
Flow cytometry gating strategy to identify fibrocytes and activated fibrocytes from whole PBMC specimens Whole PBMCs were gated on side scatter area (SSC-A) and forward scatter area (FSC-A) to gate out debris. AccuCheck counting beads are gated in the top left-hand corner. Forward scatter height (FSC-H) and FSC-A gated out doublets followed by a viability gate using LIVE/DEAD stain and FSC-A. Lymphocytes and monocytes were selected using CD45^+^ followed by gating for CD11b^+^ cells. Classical (CD14^+^/CD16^‒^, no. 3), intermediate (CD14^+^/CD16^‒^, no. 2), and nonclassical (CD14^lo^/CD16^+^, no. 1) monocyte fractions were determined next, with each portion examined for fibrocytes. Collagen type 1 (COL-1)–positive cells (no. 5) were further examined for α-SMA positivity to determine activation status (no. 4).

**FIGURE 2.**
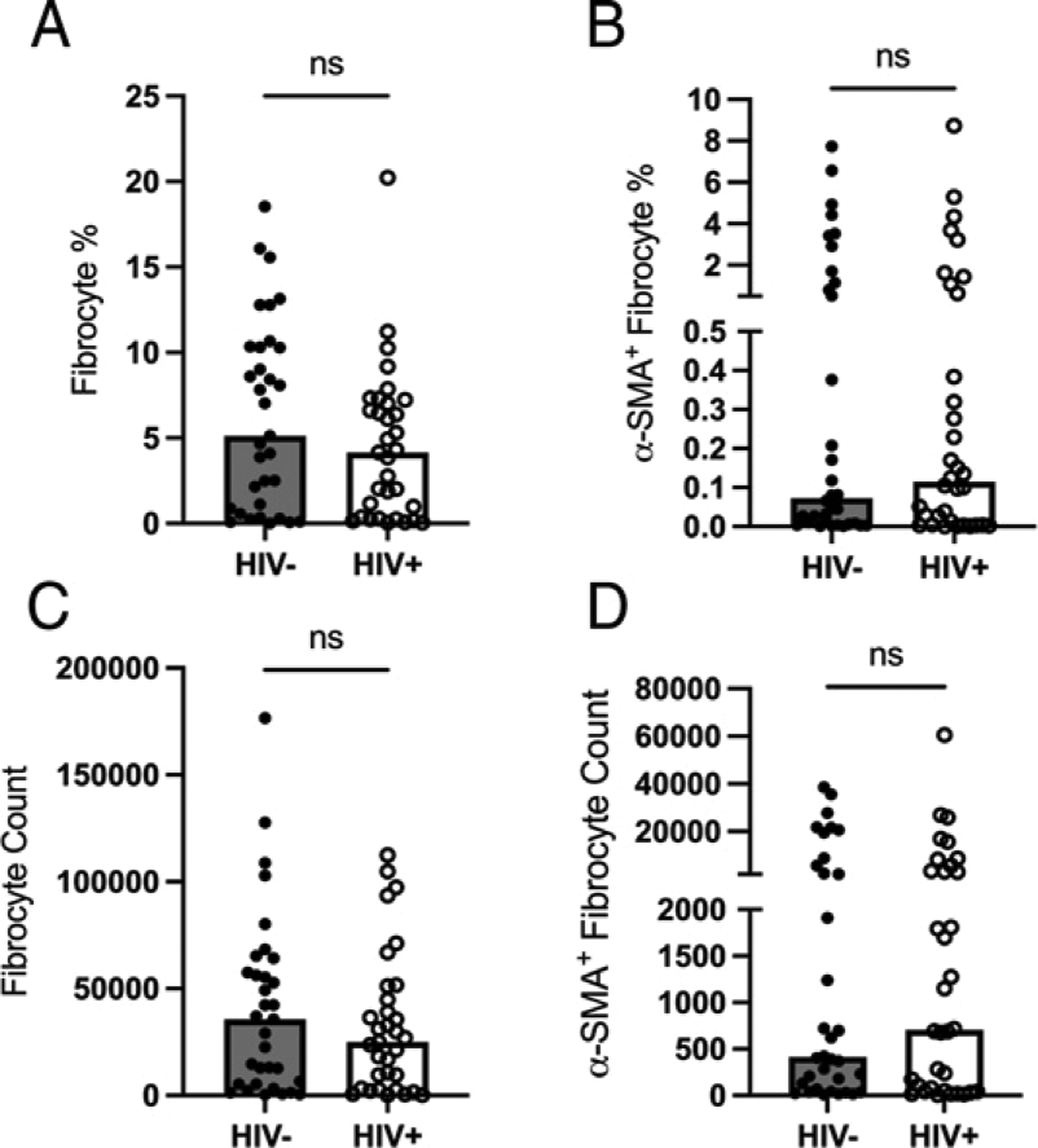
**(A–D)** Comparison of percentage of fibrocytes from CD45^+^ cells (A), percentage of activated fibrocytes from CD45^+^ cells (B), total fibrocyte count (C), and total activated fibrocyte count (D) between HIV‒ and HIV+ groups. Significance was determined by a Mann–Whitney *U* test. ns, not significant.

To identify and compare circulating fibrocyte levels between HIV‒ and HIV+ groups, PBMCs were stained with fluorescent-conjugated Abs (CD45, CD11b, CD14, CD16, COL-1, and α-SMA). A representative dot plot of the gating strategy for identifying circulating fibrocytes is shown in Fig. 1. In line with previous findings (26, 30–35), our data demonstrated that circulating fibrocytes compromise ~1–5% of CD45^+^ cells (Fig. 2A) in HIV‒ individuals. Neither the percentage of fibrocytes nor the total fibrocyte counts differed significantly between the two groups (Fig. 2A, 2C).

It is known that fibrocytes differentiate into α-SMA-expressing myofibroblast-like cells in both in vitro and animal models (36, 37, 39). We therefore examined α-SMA expression on fibrocytes (referred to hereafter as differentiated fibrocytes) to determine whether circulating fibrocytes from HIV+ individuals have an increased capacity for differentiation. Compared to the HIV‒ group, the percentage and number of α-SMA^+^ fibrocytes trended higher in the HIV+ group (0.115 versus 0.073% [0.946] and 705 cells versus 413 cells [0.985], respectively), although the difference was not significant (Fig. 2B, 2D), likely the result of a large data variance.

### The composition of fibrocytes derived from monocyte subsets is similar between HIV+ and HIV‒ individuals

**FIGURE 3.**
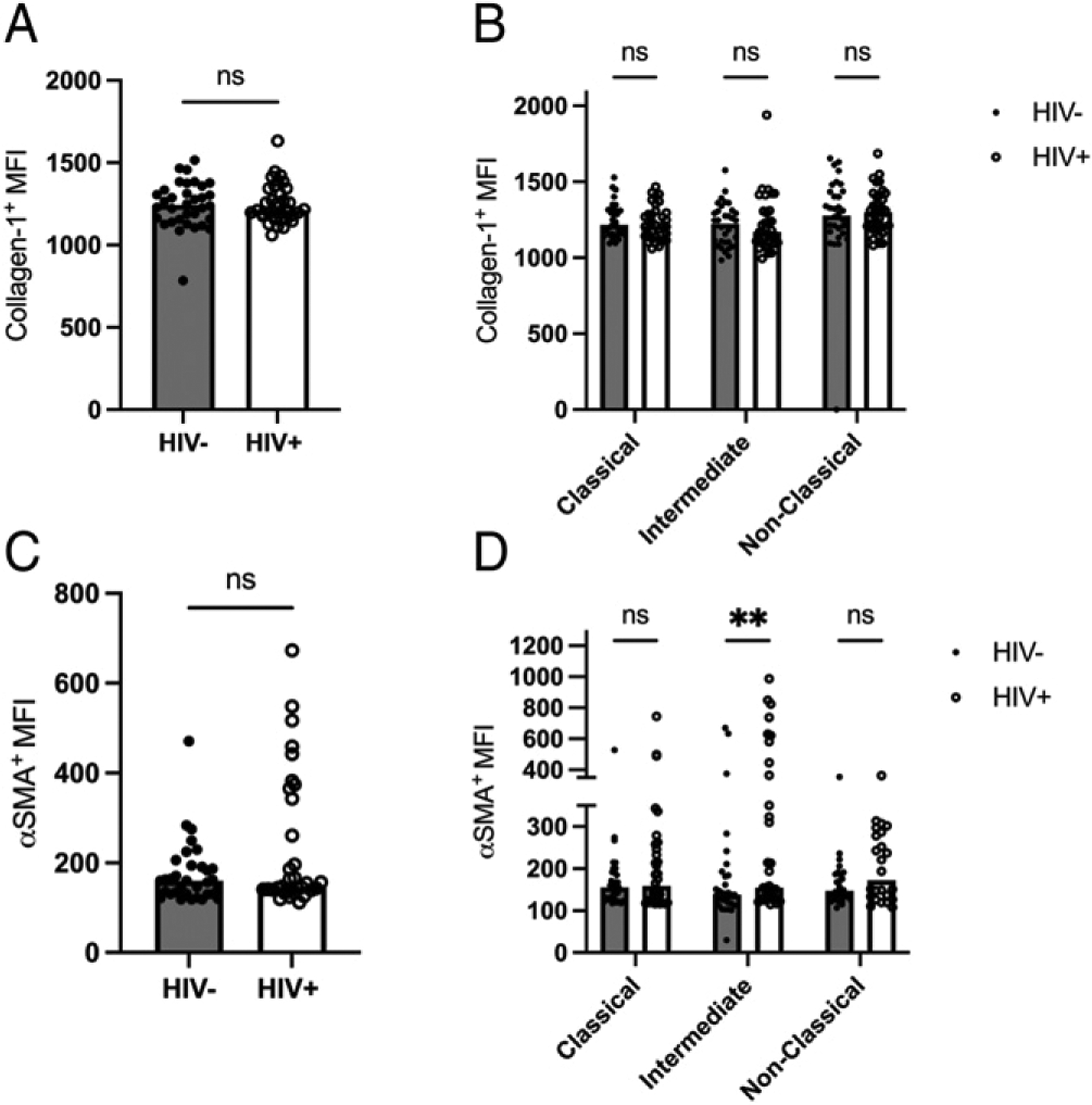
**(A)** Median fluorescent intensity (MFI) of collagen-1 from total fibrocytes in HIV‒ and HIV+ participants. (**B**) MFI of collagen-1 stratified into fibrocytes from classical, intermediate, and nonclassical monocyte lineages in HIV‒ and HIV+ participants. (**C**) MFI of α-SMA from total fibrocytes in HIV‒ and HIV+ participants. (**D**) MFI of α-SMA stratified into fibrocytes from classical, intermediate, and nonclassical monocyte lineages in HIV‒ and HIV+ participants. Significance was determined by a Mann–Whitney *U* test. **p < 0.01. ns, not significant.

Fibrocytes have classically been attributed to arise from CD14^+^ monocyte precursors (30, 38, 40, 41). Classical monocytes account for most of the total monocyte populations (80–90%), but small proportions of cultured fibrocytes have been demonstrated to express CD16, an intermediate and nonclassical monocyte marker (42). Therefore, we further investigated the similarities and differences of the composition of fibrocyte populations derived from monocyte subsets between the HIV+ and HIV ‒groups. In agreement with previous findings (43), our data demonstrate that most (~98%) fibrocytes are identified from classical monocytes (Supplemental Fig. 4). In addition, the composition of fibrocytes derived from monocyte subsets was similar between the two groups (Supplemental Fig. 4). To characterize the circulating fibrocyte phenotype and differentiation in HIV, we next examined the expression of COL-1 and α-SMA in fibrocytes. The median fluorescence intensity (MFI) of COL-1 in fibrocytes did not differ between HIV+ and HIV‒ (Fig. 3A), even when stratified according to the originating monocyte subsets (Fig. 3B). Interestingly, the MFI of α-SMA was not significantly elevated in total HIV+ circulating fibrocytes (Fig. 3C) but it was significantly elevated in fibrocytes derived from intermediate monocytes (Fig. 3D). No difference in intermediate monocyte counts (Supplemental Fig. 3E) was observed between the HIV+ and HIV‒ groups.

### Fibrocyte populations are increased in relationship to increased age

**FIGURE 4.**
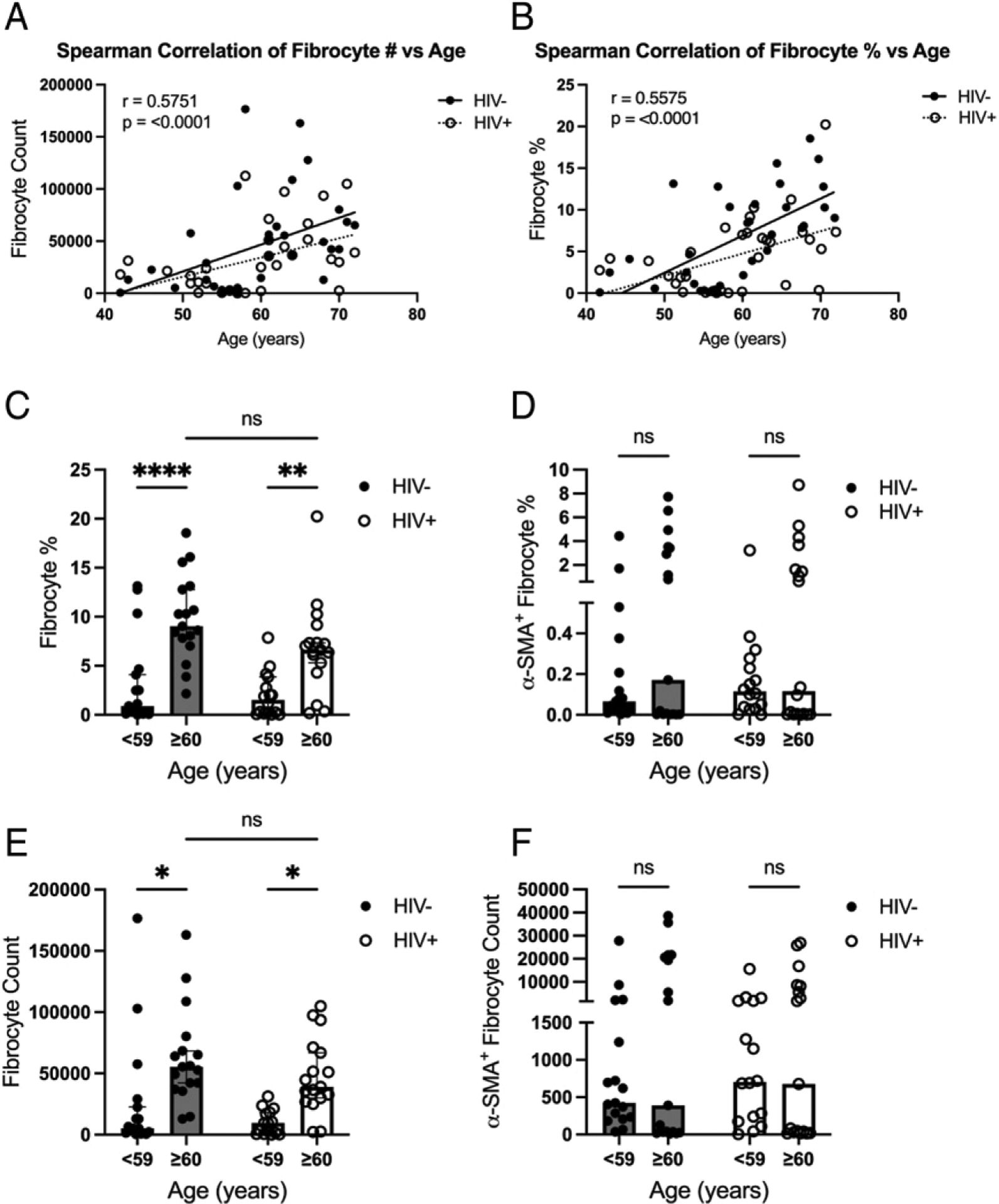
(**A** and **B**) Spearman correlation with linear regression for line of best fit of fibrocyte number (A) and fibrocyte percentage of CD45^+^ cells (B) versus age. (**C–F**) Comparison of percentage of fibrocytes from CD45^+^ cells (C), total fibrocyte count (D), percentage of activated fibrocytes from CD45^+^ cells (E), and total activated fibrocyte count (F) between HIV‒ and HIV+ groups stratified into <59 and ≥60 y of age. Significance was determined by a Mann–Whitney *U* test. *p < 0.05, **p < 0.01, ****p < 0.0001. ns, not significant.

PLWH experience age-related comorbidities at an increased prevalence compared with the HIV‒ general population. Studies have shown that circulating fibrocytes increase during normal aging (44), but this observation has not been examined in people aging with HIV. Our results demonstrate that the percentage and number of fibrocytes are positively associated with increasing age in both the HIV‒ and HIV+ groups (*p* < 0.0001) (Fig. 4A, 4B). To compare circulating fibrocyte and differentiated fibrocyte levels between HIV‒ and HIV+ based on age, study participants were divided into two age groups: group 1, <59 y old and group 2, ≥60 y old. The percentage and number of fibrocytes were significantly increased in the ≥60-y-old HIV+ and HIV‒ age groups when compared with the <59-y-old HIV+ and HIV‒ age groups (*p* ≤ 0.0001 and *p* = 0.006, respectively) (Fig. 4C, 4E). In addition, HIV+ individuals <59 y of age appeared to show an increase in both the percentage and number of α-SMA^+^ fibrocytes, compared with HIV‒ individuals (Fig. 4D, 4F). Accordingly, the median value was similar between HIV+ individuals <59 y of age and those ≥60 y of age (0.12 versus 0.37% [*p* = 0.695] and 705 versus 1814 cells [*p* = 0.597], respectively) (Fig. 4D, 4F).

## DISCUSSION

We hypothesized that the chronic inflammatory environment present in PLWH would result in increased circulating fibrocyte levels and skew fibrocytes toward a differentiated phenotype compared with HIV-seronegative controls. Our data do not support this initial hypothesis, as the percentage and number of circulating fibrocytes in PLWH are similar to HIV‒ participants. However, there was a trend of increases in both the percentage and number of differentiated fibrocytes in HIV+ compared with HIV‒ The similar baseline demographics and NICM prevalence of both groups suggests a similar health status of HIV+ and HIV‒ participants. This similarity in baseline health status allows a comparison between groups while controlling for alternative factors besides HIV status. Our HIV+ participants had a median of 19.5 y of sustained ART and were virally suppressed. These factors are plausible reasons for the similar circulating fibrocyte levels between two groups.

Identification of fibrocytes from nonclassical and intermediate monocyte populations was intended to account for alterations of monocyte subsets, previously described in PLWH, that may impact observed fibrocyte populations (45). Although no differences in fibrocyte proportions derived from monocyte subsets were observed between groups, interestingly, HIV individuals showed higher levels of α-SMA MFI in intermediate monocyte-derived fibrocytes. These data suggest that this fibrocyte subset exhibits an increasingly activated phenotype in individuals with HIV. The proinflammatory phenotype of intermediate monocytes is well established and is associated with cardiovascular disease in PLWH, independent of viral suppression and traditional cardiovascular disease risk factors (46). Despite their small population, further examinations into intermediate monocyte-derived fibrocytes and their activation state in PLWH may be warranted.

Although our data do not support enhancement of fibrocyte populations in PLWH as compared with HIV‒ participants, our results are consistent with previous reports regarding increased fibrocytes during aging (31), as we also found that fibrocyte numbers and percentages are significantly associated with aging in both HIV+ and HIV‒ participants. In addition, age-related changes in circulating fibrocyte levels were similar in both HIV+ and HIV‒ individuals. HIV infection has been associated with earlier onset of age-related NICM incidence (17, 47, 48). Interestingly, the HIV‒ group appears to exhibit an increased correlation of fibrocyte number versus age (*r* = 0.600 and *r* = 0.546, respectively) and fibrocyte percentage versus age (*r* = 0.621 and *r* = 0.521, respectively) compared with the HIV+ group. This suggests that fibrocyte population and age associations exhibit an increased trend within HIV‒ groups, compared with HIV+ groups. It is possible that the well-controlled nature of the PLWH participants exhibits a protective effect on age-related fibrocyte population increases. Examination of nonsuppressed PLWH or HIV+ individuals with fewer years of ART would likely reveal increased associations between age and fibrocyte populations.

This study has several limitations that may influence our findings. A small sample size (68 participants) was available for study based on specimen availability. Studies in larger cohorts would be more effective at determining whether fibrocytes and their phenotype are different. In addition, larger sample sizes would clarify whether increasing trends of differentiated fibrocyte populations and their activation in HIV+ participants observed in our study are sustained within large-scale cohorts. Our observations likely do not accurately represent fibrocyte populations in PLWH with uncontrolled HIV infection. Future studies are needed to determine whether circulating fibrocytes are increased in uncontrolled HIV infection and whether viral load influences circulating fibrocyte numbers and activation. As development of therapeutics for fibrosis aims to reduce populations of cellular mediators, such as fibrocytes, further studies will be needed to evaluate whether circulating fibrocyte levels are associated with biomarkers for fibrosis and the presence of fibrosis in NICMs.

Taken together, we demonstrate that well-controlled PLWH with nearly 20 y of ART adherence have similar levels of circulating fibrocytes compared with HIV‒ individuals. In addition, our findings corroborate previous observations that aging significantly increases fibrocyte populations within individuals, regardless of HIV status.

## Supplementary Material

Supplemental Figure 3

Supplemental Figure 2

Supplemental Figure 4

Supplemental Figure 1

## Figures and Tables

**TABLE I.** Demographics, noninfectious comorbidities, laboratory parameters, immunologic parameters, and hematologic parameters in HIV+ and HIV‒ participants

		HIV+ (*n* = 34)	HIV− *(n =* 34)
Demographics	Age (y)	59.9 [53.2–64.9]	59.2 [54.4–65.0]
	Male (*n*, %)	30 (88.2)	30 (88.2)
	White (*n*, %) Body mass index (kg/m^2^)	22 (64.7)	21 (61.8)
		26.1 [23.1–28.8]	28.6 [23.7–32.6]
Comorbidities	Diabetes (*n*, %)	5 (14.7)	4 (11.8)
	Hypertension (*n*, %)	13 (38.2)	13 (38.2)
	High cholesterol (*n*, %)	16 (47.1)	12 (35.3)
	History of smoking (*n*, %)	20 (58.8)	17 (50.0)
	COPD (*n*, %)	2 (5.9)	1 (2.9)
	Asthma (*n*, %)	8 (23.5)	9 (26.5)
	GI problem (*n*, %)	11 (32.4)	13 (38.2)
	Hepatitis B (*n*, %)	3 (8.8)	0 (0)
	Hepatitis C (*n*, %)	2 (5.9)	3 (8.8)
	Chronic kidney disease (*n*, %)	5 (14.7)	2 (5.9)
Laboratory parameters	Cholesterol (mg/dl)	168.5 [145.0–199.3]	179.5 [159.3–201.5]
	Triglycerides (mg/dl)	113.5 [80.5–153.8]	89.0 [74.5–130.3]
	LDL-C (mg/dl)	106.0 [68.0–140.0]	114.5 [96.8–140.0]
	HDL-C (mg/dl)	45.0 [38.5–52.3]	48.5 [42.0–57.5]
	AST (U/l)	23.0 [18.5–28.5]	21.0 [17.0–28.0]
	ALT (U/l)	24.5 [14.8–34.3]	18.0 [14.8–30.5]
	Systolic BP (bpm)	127.0 [119.8–133.0]	132.0 [119.3–142.3]
	Diastolic BP (bpm)	77.5 [69.5–85.3]	78.5 [71.8–86.5]
HIV immunologic parameters	Lowest recorded CD4^+^ T cell count (cells/μl)	200.0 [85.8–431.3]	—
	ART (y)	19.5 [10.5–24.5]	—
	Entry CD4^+^ T cell count (cells/μl)	747.5 [569.0–926.5]	—
Hematological parameters	Absolute lymphocyte count (×10^3^/μl)	2.0 [1.7–2.4]	1.7 [1.4–2.1]*
	Absolute monocyte count (×10^3^/μl)	8.7 [7.2–9.7]	8.5 [6.9–9.7]
	Absolute platelet count (×10^9^/l)	218.5 [187.8–258.3]	233.0 [200.0–279.8]
	Absolute neutrophil count (×10^3^/μl)	2.9 [2.3–3.9]	3.7 [2.5–4.5]

Values are presented as median [Quartile 1–Quartile 3] unless otherwise stated.

Dashes denote no data, as these tests/medication years, or CD4 counts are not tracked for HIV‒ participants.

Statistical significance was determined by a Mann–Whitney U test.

* *p* < 0.05.

ALT, alanine aminotransferase; AST, aspartate aminotransferase; bpm, beats per minute; COPD, chronic obstructive pulmonary disease; GI, gastrointestinal.

## Abbreviations used in this article:

ART: antiretroviral therapy
BP: blood pressure
COL-1: collagen type 1
DPBS: Dulbecco’s PBS
HDL-C: high-density lipoprotein cholesterol
LDL-C: low-density lipoprotein cholesterol
MFI: median fluorescence intensity
NICM: noninfectious comorbidity
PLWH: people living with HIV
RT: room temperature
a-SMA: a-smooth muscle actin
