# Supplementary figures and images for: Characterization of Circulating Fibrocytes in People Living with HIV on Stable Antiretroviral Therapy
Source: Immunohorizons. Author manuscript; Available in PMC 2023 Aug 4. (PMC10402248; doi:10.4049/immunohorizons.2200085)

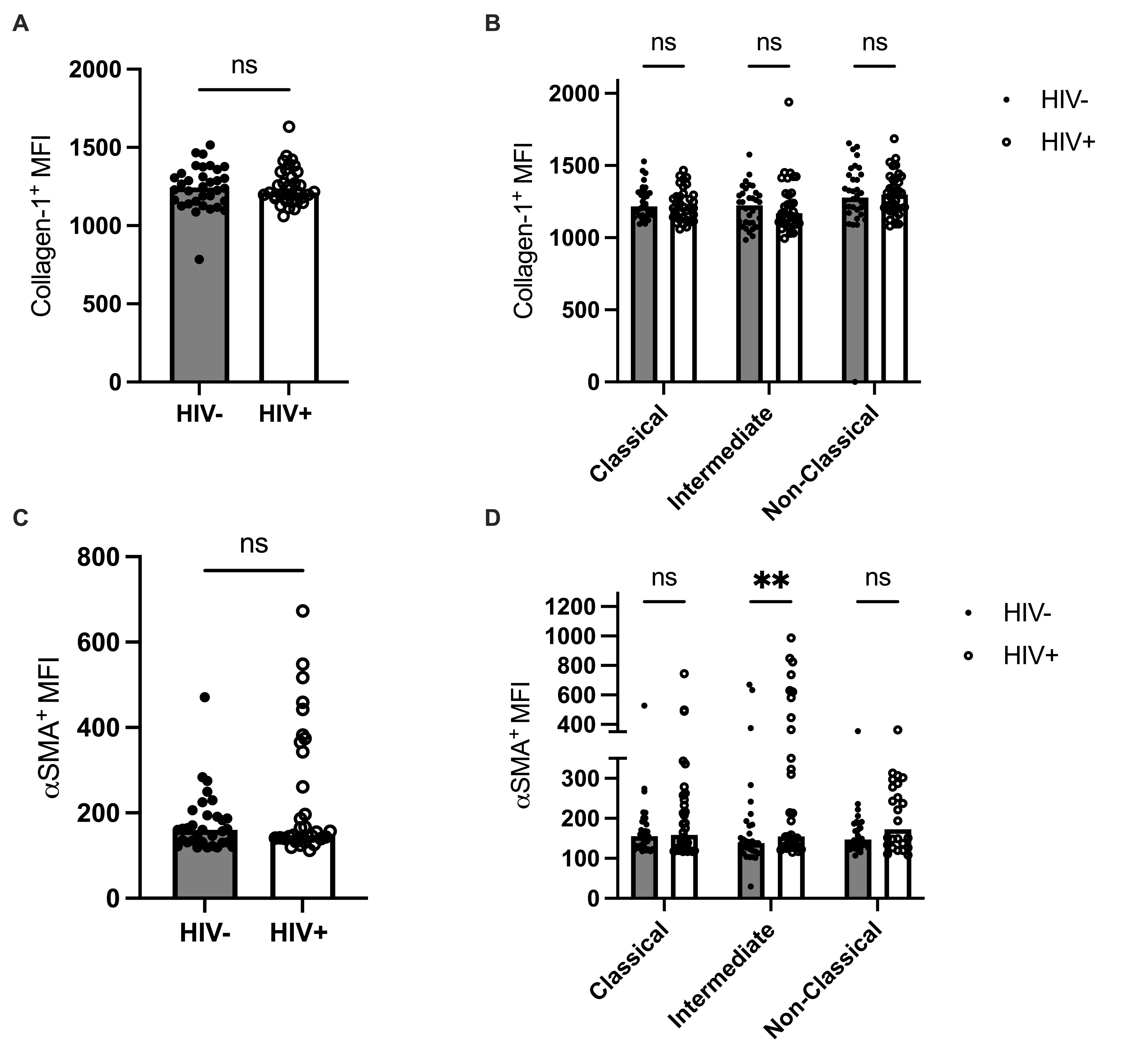

Supplement: Supplemental Figure 3 [file NIHMS1921299-supplement-Supplemental_Figure_3.tiff]

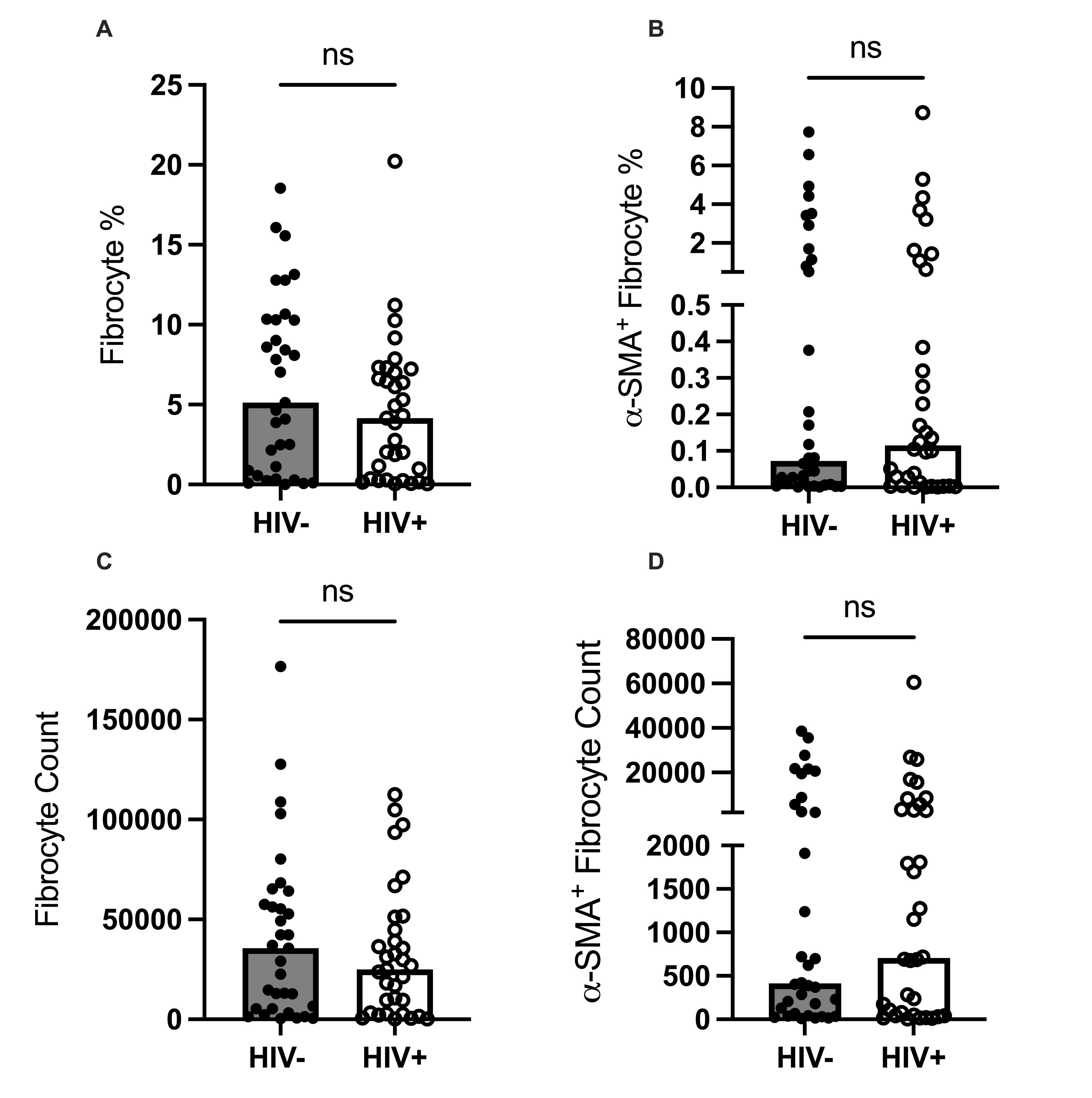

Supplement: Supplemental Figure 2 [file NIHMS1921299-supplement-Supplemental_Figure_2.tiff]

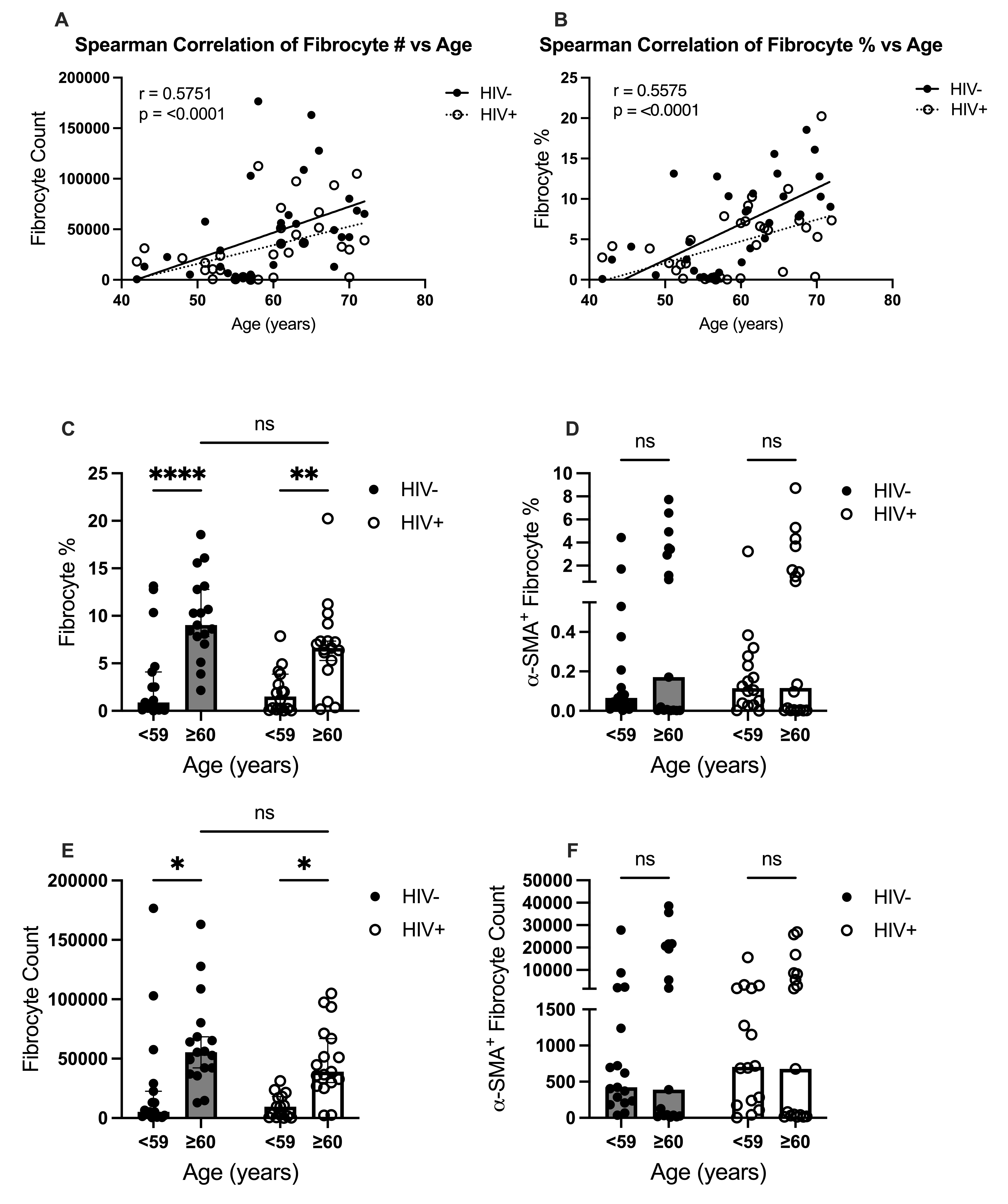

Supplement: Supplemental Figure 4 [file NIHMS1921299-supplement-Supplemental_Figure_4.tiff]

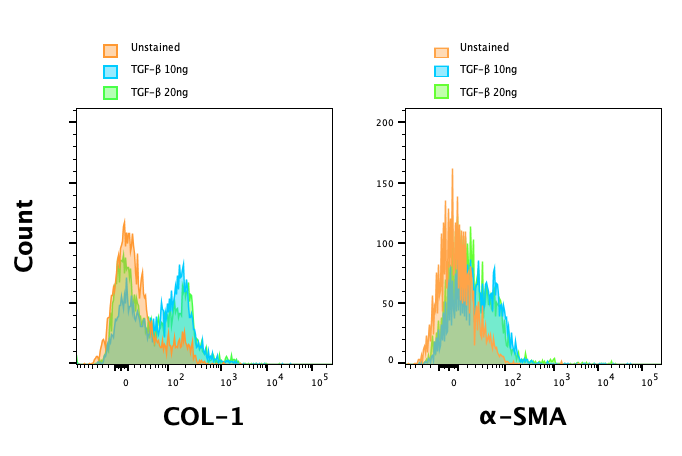

Supplement: Supplemental Figure 1 [file NIHMS1921299-supplement-Supplemental_Figure_1.tiff]
